# Post-zygotic sterility and cytonuclear compatibility limits in *S. cerevisiae* xenomitochondrial cybrids

**DOI:** 10.3389/fgene.2014.00454

**Published:** 2015-01-12

**Authors:** Mário Špírek, Silvia Poláková, Katarína Jatzová, Pavol Sulo

**Affiliations:** Department of Biochemistry, Faculty of Natural Sciences, Comenius UniversityBratislava, Slovakia

**Keywords:** cytonuclear incompatibility, xenomitochondrial cybrids, *Saccharomyces* yeasts, adaptation, Dobzhansky–Muller pairs, speciation

## Abstract

Nucleo-mitochondrial interactions, particularly those determining the primary divergence of biological species, can be studied by means of xenomitochondrial cybrids, which are cells where the original mitochondria are substituted by their counterparts from related species. *Saccharomyces cerevisiae* cybrids are prepared simply by the mating of the ρ^0^ strain with impaired karyogamy and germinating spores from other *Saccharomyces* species and fall into three categories. Cybrids with compatible mitochondrial DNA (mtDNA) from *Saccharomyces paradoxus* CBS 432 and *Saccharomyces cariocanus* CBS 7994 are metabolically and genetically similar to cybrids containing mtDNA from various *S. cerevisiae.* Cybrids with mtDNA from other *S. paradoxus* strains*, S. cariocanus, Saccharomyces kudriavzevii, and Saccharomyces mikatae* require a period of adaptation to establish efficient oxidative phosphorylation. They exhibit a temperature-sensitive phenotype, slower growth rate on a non-fermentable carbon source and a long lag phase after the shift from glucose. Their decreased respiration capacity and reduced cytochrome *aa3* content is associated with the inefficient splicing of *cox1*I3β, the intron found in all *Saccharomyces* species but not in *S. cerevisiae.* The splicing defect is compensated in cybrids by nuclear gain-of-function and can be alternatively suppressed by overexpression of *MRP13* gene for mitochondrial ribosomal protein or the *MRS2, MRS3*, and *MRS4* genes involved in intron splicing. *S. cerevisiae* with *Saccharomyces bayanus* mtDNA is unable to respire and the growth on ethanol–glycerol can be restored only after mating to some *mit*^−^ strains. The nucleo-mitochondrial compatibility limit of *S. cerevisiae* and other *Saccharomyces* was set between *S. kudriavzevii* and *S. bayanus* at the divergence from *S. cerevisiae* about 15 MYA. The *MRS1-cox1 S. cerevisiae*/*S. paradoxus* cytonuclear Dobzhansky–Muller pair has a neglible impact on the separation of species since its imperfection is compensated for by gain-of-function mutation.

## Introduction

Understanding the genetic basis of speciation is one of the major tasks in evolutionary biology. In spite of the recent methodological progress, many questions still remain unanswered or debated (Nosil and Schluter, 2011; Butlin et al., 2012). In general several types of reproductive isolation have been considered, but most genetic studies are focused on the speciation process derived from interspecific hybrids. Consequently, the cause of the inviability and sterility resulting from the post-zygotic barrier has been studied (Dobzhansky, 1937; for a review see Butlin et al., 2012). Yeasts, particularly *Saccharomyces cerevisiae*, are the experimental model of choice for such speciation studies due to their ability to mate with related species and their homothallism, allowing the mating type switch in haploid cells after almost every division. Therefore, interspecies hybrids between different *Saccharomyces* species are able to establish fertile lines of “a new species” after repeated cycles of sporulation and self-fertilization (Greig et al., 2002; reviewed in Greig, 2009; Chou and Leu, 2010; Louis, 2011; Morales and Dujon, 2012). In *Saccharomyces* yeasts, the reproductive barrier is post-zygotic and this can be attributed to non-collinear chromosomes' (Delneri et al., 2003; Charron et al., 2014; Hou et al., 2014) “anti-recombination” resulting from the action of a “confused” mismatch repair system (Hunter et al., 1996; for a review see Greig, 2009; Louis, 2011).

In general, the divergence of species results from responses to negative epistatic genetic interactions, known as “Dobzhansky–Muller incompatibilities,” which are alleles that are completely normal in their native genetic background but are deleterious on the genetic background of the other population. The hypothesis was confirmed experimentally and reduced efficiency of meiotic reproduction was observed in hybrids made from populations adapted to two divergent environments (Dettman et al., 2007) and later some genes responsible for the divergence were identified (Anderson et al., 2010). So far, no Dobzhansky–Muller nuclear incompatibilities have been found in yeasts (Greig, 2007; Kao et al., 2010), but they have been reported between mitochondrial and nuclear genes (reviewed in Lee et al., 2008; Chou and Leu, 2010; Chou et al., 2010).

Functional mitochondria are indispensable for yeast sporulation, which is the essential step in hybrid speciation. The fate of mitochondrial DNA (mtDNA) has been largely overlooked in hybrid speciation lines, but it is believed that progeny inherit a complete mitochondrial (mt) genome from one of the yeast species, as has been described in natural or *in vitro* created hybrids (Marinoni et al., 1999; Rainieri et al., 2008; Solieri et al., 2008). Mitochondria have a limited genome, and efficient oxidative phosphorylation requires co-adaptation of mitochondrial and nuclear genes, despite divergent paces of evolution. Their biogenesis depends on the tender interplay of up to thousand proteins encoded by the nucleus, with eight proteins and several dozen gene products encoded by *Saccharomyces* mtDNA (for a review see Lipinski et al., 2010; Schmidt et al., 2010; Fox, 2012). Consequently, in interspecific hybrids the mitochondrial genome from one partner does not have to communicate equally well with the nuclear genome of the second partner and the progeny can be sterile or non-viable. Such nucleo-mitochondrial communication has been reported as a determinant of reproductive isolation during yeast evolution (Lee et al., 2008; Chou et al., 2010; reviewed in Chou and Leu, 2010; Solieri, 2010). Pairs of genes with interspecific incompatibility were found using non-respiring chimeras containing mitochondria from one partner as well as a set of original chromosomes, where one or two were replaced with their counterpart from second partner (Lee et al., 2008; Chou et al., 2010). Then, the elements of interspecific nucleo-mitochondrial incompatibility can be recovered from genomic DNA libraries according to the ability to rescue the respiratory defect. This approach has revealed several Dobzhansky–Muller pairs: (i) *Saccharomyces bayanus* nuclear *AEP2* allele with *S. cerevisiae/Saccharomyces paradoxus* mitochondrial *atp9* gene; (ii) *S. cerevisiae MRS1* nuclear gene required for the splicing of some mitochondrial group I introns with *S. paradoxus/S. bayanus* mtDNA likely some in *cox1* genes; and (iii) *S. cerevisiae/S. paradoxus AIM22* gene variants coding enzyme required for the addition of lipoate to some mitochondrial proteins imported from cytoplasm with *S. bayanus* mitochondria (Chou et al., 2010). In spite of providing valuable data, this approach failed in the preparation of an entire set of chimeras with single chromosome replacement implicating additional Dobzhansky–Muller pairs (Lee et al., 2008; Chou et al., 2010).

Dobzhansky–Muller cytonuclear incompatibilities can generally be directly studied by the transplacement of mitochondria from one species to mutants lacking mitochondrial DNA (ρ^0^) from a second species, according to the restoration of the ability to respire and grow on a non-fermentable carbon source. In early studies mitochondria from unrelated yeast species were introduced to *S. cerevisiae* (Yoshida, 1979; Sakanaka et al., 1996; Osusky et al., 1997). However, these experiments could not be reproduced (Špírek et al., 2000) and most of them should be considered experimental artifacts, mainly as consequence of the absence of mtDNA analysis in cybrids and unreliable taxonomy, as most of the “unusual” compatible yeasts were misclassified (Barnett, 1992; Špírek et al., 2000; Vaughan-Martini and Martini, 2011; Sulo, unpublished results). Currently, the *Saccharomyces* genus consists of the species *S. cerevisiae*, *S. paradoxus, Saccharomyces mikatae, Saccharomyces kudriavzevii, S. arboricolus, S. bayanus* var. *bayanus*, *S. bayanus* var. *uvarum*, and *Saccharomyces pastorianus* (Vaughan-Martini and Martini, 2011). However, whole genome analysis has shown that the last three species are cold-adapted alloploids of *S. cerevisiae* and the newly described species *Saccharomyces eubayanus* (reviewed in Hittinger, 2013). In addition, *Saccharomyces cariocanus* is considered to be more a *S. paradoxus* variant as this species is reproductively isolated from *S. paradoxus* by four translocations but not by sequence (Liti et al., 2006; reviewed in Hittinger, 2013). Consequently the successful re-establishment of mitochondrial functions in *S. cerevisiae* ρ^0^ strains has been achieved by the transplacement of isolated mitochondria or protoplast fusion only from yeasts currently classified as synonymous to *S. cerevisiae* (Osusky et al., 1997; Špírek et al., 2000) or the most closely-related species *S. paradoxus* (Špírek et al., 2000). However, in this case the rate of the re-established respiration was only partial (Špírek et al., 2000). In the opposite direction, *S. cerevisiae* mtDNA was able to restore respiration in *S. paradoxus* mitochondria to a level close to the original (Sulo et al., 2003), indicating the unidirectional character of these nucleo-mitochondrial incompatibilities (Sulo et al., 2003), a phenomenon that has been confirmed in interspecific hybrids with different combinations of nuclear and mitochondrial genomes (Chou et al., 2010).

Thus, xenomitochondrial cybrids are a valuable tool in the study of nucleo-mitochondrial interactions, particularly those determining the primary divergence of biological species. In this work we elaborated a simple procedure for the preparation of xenomitochondrial cybrids with the aim to report nucleo-mitochondrial incompatibilities associated with an inability to splice an unusual mitochondrial intron *cox1*I3β and the capability of cells to compensate for the splicing defect after the adaptation period by the gain-of-function of nuclear genes.

## Materials and methods

### Yeast strains

The *Saccharomyces* species used for the transfer of mitochondria to *S. cerevisiae* were as follows: *S. cerevisiae* NRRL Y-12632^*T*^ (CBS 1171), *S. paradoxus* NRRL Y-17217^*T*^ (CBS 432), *S. paradoxus* (CBS 2908), *S. kudriavzevii* NRRL Y-27339^*T*^ (CBS 8840), *S. cariocanus* NRRL Y-27337^*T*^ (CBS 7994), *S. mikatae* NRRL Y-27341^*T*^ (CBS 8839), *S. bayanus* var. *uvarum* NRRL Y-17034^*T*^ (CBS 395), *S. bayanus* var. *bayanus* NRRL Y-12624^*T*^ (CBS 380), and *S. pastorianus* (CBS 1504, CBS 1513), which were obtained from J. Piškur Lund University. The following abbreviations apply: CBS corresponds to the Culture Collection of the Centraalbureau voor Schimmelcultures and Fungal Biodiversity Center, Utrecht, The Netherlands; NRRL to the Agricultural Research Service Culture Collection, US Department of Agriculture, Peoria, Illinois, USA; and NCYC to the National Collection of Yeast Cultures, Institute of Food Research, Norwich, United Kingdom. The superscript *T* in yeast designations indicates type of strains (Vaughan-Martini and Martini, 2011). The other *S. cerevisiae* wild type strains used in this study are as follows: CCY 21-37-2 (CBS 457), CCY 21-46-1 (CBS 4054), CCY 21-37-1 (CBS 1426), CCY 21-45-1 (CBS 1782), CCY 21-8-1 (CBS 436), CCY 21-4-11 (CBS 1460), CCY 21-11-1 (formerly *Saccharomyces chevalieri*, CBS 400), CCY 21-10-1 (CBS 435), CCY 21-36-1, CCY 21-42-1 (formerly *Saccharomyces capensis*, CBS 2247), CCY 21-33-3 (formerly *Saccharomyces italicus*, CBS 2909), CCY 21-21-1 (formerly *Saccharomyces oviformis*, CBS 429), CCY 21-14-1, CCY 21-1-1 (CBS 382), CCY 21-4-27 (CBS 1200), CCY 21-15-6 (CBS 439), and CCY 21-15-1 (CBS 381), which were obtained from the Culture Collection of Yeasts (CCY; the former Czechoslovak Collection of Yeasts), located at the Institute of Chemistry, Slovak Academy of Sciences, in Bratislava. The proper taxonomic classification of any strain used in this study has been confirmed by the sequencing of the D1/D2 region from a large ribosomal RNA subunit and mitochondrial *cox2* and *rns* genes, as described in Kurtzman (2003). *S. paradoxus* (synonymous to *Saccharomyces douglasii*) CBS 7400 strain was kindly provided by H. Fukuhara, Institute Curie, Orsay. The laboratory acceptor strains of mitochondria MCC109 ρ^0^ (*MATα, ade2-1, ura3-52, kar1-1*, ρ^0^); *mit*^−^ testers PTY6 (*MATα, ade2, ura3-52, kar1-1, cox2-17*), K2145 (*MATa, ade2, lys, kar1-1, cox2-17*), 2612 [*MATα, ade1, op1, mit^−^(cox1 ΔB)]*, MD 79 [*MAT a, leu1, kar1-1* (*cox1-delB*)], akar170 [*MATa, his^−^, lys^−^, kar1-1, syn^−^* (*trnD C→A* (*72*)], V281 [*MATα, ade1, op1, mit^−^* (*cox1, aI1 T→A*(*1856*)], αOP1/M1301 [*MATα, op1, ura3-1, leu2-3, 112, his3-11,15, trp1-289, mit^−^* (*cob bI1M1301*)], as well as derivatives of W303 1A *(MATa, ade2-1, trp1-1, leu2-3, 112, his3-11,15, ura3-1, can1-100, Gal^+^, psi*^+^, ρ^+^), DBY 747 *(MATa, trp1-289, leu2-3, 112, his3-1, ura3-52, Gal^−^*, ρ^+^), and DBY 747/M1301 [*MATa, trp1-289, leu2-3, 112, his3-1, ura3-52, Gal^−^, mit*^−^ (*cob bI1M1301*)] have been described by Špírek et al. (2000, 2002) and Sulo et al. (2003). Other *mit*^−^ strains 5B [*MATa, ade1^−^, lys1^−^, mit^−^ (cox1 ai1ai2 junction)*], and AD1 [*MATa, ade1^−^, lys1^−^, mit^−^ (cox1 ai1ai2 junction)*] are described in detail in Anziano et al. (1990).

#### Media

Yeasts were routinely cultivated on YPD (1% bactopeptone, 1% yeast extract, 2% glucose), YPGE (1% bactopeptone, 1% yeast extract, 3% glycerol, 2% ethanol), minimal medium (0.67% YNB, 2% glucose), and sporulation media (0.1% yeast extract, 1% potassium acetate, 0.05% glucose).

#### Transplacement of mitochondria by interspecific cytoduction

Sporulated diploid collection yeasts (10^8^–10^9^/50 μl) were treated with Zymolyase 20T 0.5 mg/ml. After 10–20 minutes (when the majority of spores were released), the spores were vortexed for 30 s and washed with distilled water mixed with a twofold amount of fresh MCC109 ρ^0^ cells, and the suspension was applied in a small volume (10–50 μl) on YPD plates. After one day's incubation the mating efficiency was controlled microscopically and aliquots of 10^8^ cells were streaked onto selection FOA plates (3% glycerol, 2% ethanol, 0.5% glucose, 0.67% YNB, 20 mg/l adenine, 50 mg/l uracil, 2% agar, with 1 g/l of 5-fluoroorotic acid AUDFOA) for the selection of respiring cybrids (2% glucose, 0.67% YNB, 20 mg/l adenine, 50 mg/l uracil, 2% agar, with 1 g/l of 5-fluoroorotic acid AUFOA) to select any cybrids. The plates were incubated at 28°C and after 5–7 days they were scraped and resuspended in demineralized water and spotted in tenfold dilutions onto YPD plates. After 3 days of cultivation the number of colonies grown from single drops were counted and plated in corresponding dilutions on AUFOA or YPD plates. Single colonies were then screened for correct auxotrophic markers and the ability to grow on YPGE plates. The presence of mtDNA in colonies that did not grow on YPGE plates was determined by restriction digestion or after mating to the *mit*^−^ tester strain K2145, allowing non-adapted or non-respiring cybrids to be studied. A similar selection procedure is widely used when DNA is delivered to mitochondria by biolistics (Fox et al., 1991). The approach has a few constraints, such as the poor sporulation ability observed in a number of the collection strains. However, the problem can be bypassed by several cycles of sporulation and germination. The other expected pitfall, low mating ability, was not observed since even the less related species *S. cerevisiae* and *S. bayanus* produced an incredible amount of zygotes (Marinoni et al., 1999). Finally, the acceptor strain lacking mtDNA must carry the *kar1-1* mutation, because their substitution by regular ρ^0^ strains does not yield cybrids but hybrids.

#### DNA analysis

The presence of mtDNA in cybrids was screened by the *Hinf*I restriction digestion of genomic DNA isolated according to the laboratory modification of Philippsen et al. (1991). Mitochondrial DNA was isolated according to Defontaine et al. (1991). Karyotypes have been determined by pulsed-field electrophoresis, as described in Marinoni et al. (1999), with minor modification. Electrophoresis was carried out at 150 V for 6.5 h, with a switching time of 240 s followed by 8 h with a switching time of 160 s, then 9 h 45 minutes with pulse duration of 90 s, and finally 14 h 38 minutes with 60 s pulse duration.

#### DNA amplification and sequencing

D1D2 domain of 26S rDNA was amplified according to Kurtzman and Robnett (2003), part of the mitochondrial *cox2* gene was amplified with primers cox2 357: 5′-CAG GAT CCA GCA ACA CCA AAT CAA GA and cox2 rev: 5′-CAT GCC CCA TAG AAG ACA CTT TCT CT (amplification condition 94°C – 3 min, 35 × (94°C – 30 s, 52°C – 1 min, 72°C – 1 min), 72°C – 5 min, 14°C). Part of *rns* gene was amplified with primers YM5: 5′-AAG AAT ATG TTG GTT CAG A and YM13: 5′-ATT CTA CGG ATC CTT TAA ACC A (amplification condition 94°C – 3 min 35 × (94°C – 30 s, 45°C – 1 min, 72°C – 2 min), 72°C – 5 min). *Cox1*I3β was amplified using the primers SDA2: 5′-AAT CTA CAC TAG GTC CTG AAT GTG CCT GAA and SDA3: 5′-AAT CAG GTG CTG GTA CAG GAT GAA (amplification condition 94°C – 3 min, 35 × (94°C – 30 s, 45°C – 1 min, 72°C – 2 min), 72°C – 5 min). Entire c*ox1 m*tDNA sequences were obtained as described in Prochazka et al. (2012) or were extracted from running the whole mtDNA sequencing project using Illumina MiSeq and paired-end (2 × 100 nt technology) assembled with CLC genomics Workbench 7.0.3 (http://www.clcbio.com).

#### RNA analysis

RNA was isolated as described by Köhrer and Domdey (1991). Thirty micrograms was separated on 1.0% agarose–formaldehyde gels, transferred to a nylon membrane, and hybridized by the DIG High Prime DNA Labeling and Detection Kit (Roche Molecular Biochemicals) labeled probes, as described in Brown (1993).

#### Disruption of MRP13 gene

The region between *BamHI* and *XbaI* restriction site of the gene was replaced with a *LEU2* gene *XbaI*-*BamHI* fragment and introduced to the haploid spores of adapted and non-adapted cybrids carrying the *leu2* mutation and *S. paradoxus* CBS 7400 mtDNA as well as to W303 1A ρ^0^ and ρ^+^ strains. Disruption was confirmed after Southern blotting by hybridization with *MRP13* specific probe.

#### Oxygen consumption

Oxygen consumption was measured at 30°C with a Clark oxygen electrode using 2% ethanol as the substrate. Cybrid cells were grown first for 12 h at 28°C in liquid YPD medium, then pelleted by centrifugation (10 min, 2500 g), and then cultivated an additional 12 h in 50 ml YPGE media, before the measurement cells were harvested and washed twice with distilled water. Oxygen consumption was measured in 2 ml water suspension with 2% ethanol as a substrate. Respiratory rates were expressed as consumed nmoles O_2_/min per 5 × 10^8^ cells. The solubility of the O_2_ 237 nmol/ml water was considered and at the end of measurement oxygen consumption was inhibited by the addition of antimycine at the final concentration of 10 μg/ml.

#### Cytochrome spectra

Cytochrome spectra were recorded in mitochondrial suspensions (0.4 M sorbitol, 5 mM EDTA pH 6–8, 0.25% BSA 8 mg/ml of proteins) by Double Wavelength Double Beam Perkin-Elmer 557 spectrophotometer, as described in Tzagoloff et al. (1975). Mitochondria were isolated according to (Sulo et al., 1989) from an overnight culture grown on 1% bactopeptone, 1% yeast extract, and 2% galactose. Difference spectra were recorded at room temperature in suspensions split in half, where one was oxidized by hydrogen peroxide (final concentration 50 mM) and the other was reduced by the addition of dithionite (a few crystals).

#### Growth curves

Fresh yeasts grown overnight on the plates were inoculated into liquid YPGE. At 24 h intervals the aliquots were withdrawn diluted to the density of ≤0.4 at 600 nm.

#### Preparation of ρ^−^ mutants

ρ mutations in intronless strain DBY 747 were induced by ethidium bromide, as described in Fox et al. (1991). Mutants carrying the segments of the *cox1* gene were identified according to the ability to complement *mit*^−^ mutation in *cox1* but not *mit*^−^ mutations in other genes (*cob, trnD*, *cox2*).

Yeasts were transformed according to Soni et al. (1993). Other procedures were done as described in the manuals of Ausubel et al. (2002) and Guthrie and Fink (1991).

## Results

### The ability to populate *S. cerevisiae* with mitochondria from related species—phylogenetic limit

To elucidate the limit of nucleo-mitochondrial compatibility among *Saccharomyces*, we made an attempt to construct xenomitochondrial cybrids containing nuclei from *S. cerevisiae* and mtDNA from all other *Saccharomyces* species. To avoid any misinterpretations resulting from incorrect taxonomic classification, the origin of any strain used in this study has been verified by the sequencing of the D1/D2 region from the large ribosomal RNA subunit and mitochondrial *cox2* and *rns* genes, as described in Kurtzman (2003). The collection consisted of about 20 *S. cerevisiae*, three *S. paradoxus* strains, of which two were involved in a previous study (Špírek et al., 2000), and three relatively new but quite well-characterized species: *S. cariocanus, S. kudriavzevii*, and *S. mikatae* (Naumov et al., 2000) and *S. bayanus*.

**Figure 1:**
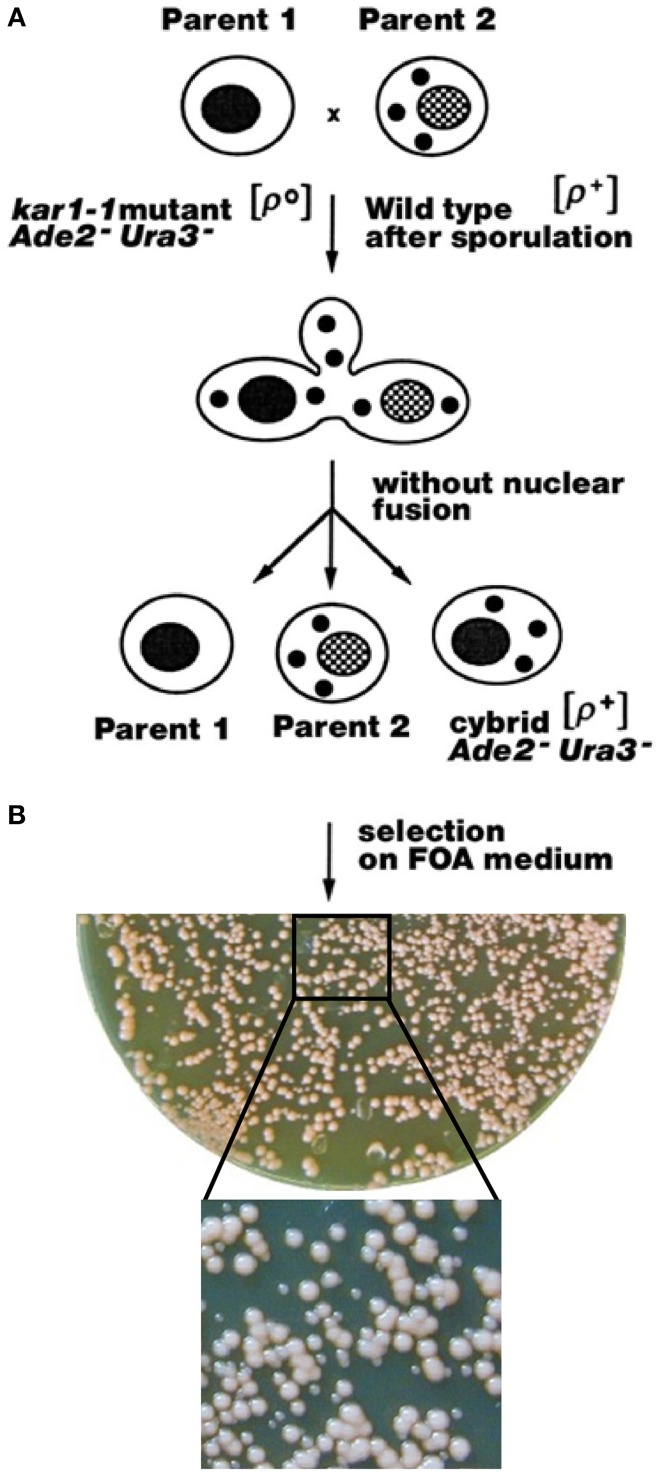
**Construction of xenomitochondrial cybrids. (A)** General selection chart. **(B)** Plating of cytoduction mixture (MCC109 ρ^0^ × *S. kudriavzevii* CBS 8840) after 5 days cultivation on selection medium with FOA on YPD plate. Detail: smaller white colonies – non-adapted cybrids; larger pink colonies – adapted cybrids.

Instead of protoplast fusion (Špírek et al., 2000) the mitochondria were transferred by mating the spores from different *Saccharomyces* species to *ade2-1, ura3-52, kar1-1*, ρ^0^ *S. cerevisiae* strain, since the *kar1-1* mutation in the acceptor strain significantly reduces the karyogamy events (Fox et al., 1991). Cybrids regaining efficient mitochondrial functions can be simply selected as the mostly red/pink colonies on the plates containing 5-fluoroorotic acid (FOA) and an excess of non-fermentable carbon source. FOA is lethal for any yeast carrying the wild type *URA3* gene (Sikorski and Boeke, 1991), and therefore it inhibits the growth of “true hybrids” and all wild type *Saccharomyces* strains (Figure 1). Cybrids with various degrees of re-established mitochondrial functions can be also obtained on synthetic selection plates with FOA and glucose as a carbon source, as they can preferentially survive 5–7 days incubation (Figure 1B). After plating to a single colony, cybrids from donor species can be distinguished according to their auxotrophy.

**Figure 2:**
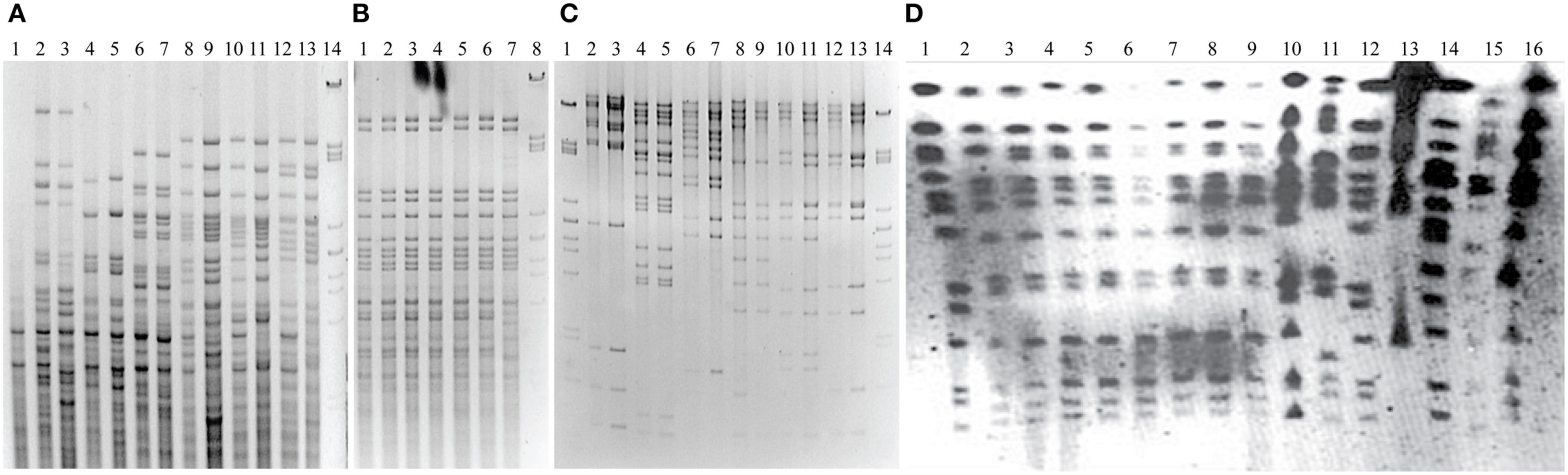
**Origin of mitochondrial and nuclear genomes in cybrids. (A)** *Hinf*I digest of genomic DNA separated on 1% agarose gel. Lanes: *S. cerevisiae* MCC109 ρ^0^ (acceptor strain) (1); *S. kudriavzevii* CBS 8840 cybrid (2) parent (3); *S. mikatae* CBS 8839 cybrid (4) parent (5); *S. cariocanus* CBS 7994 cybrid (6) parent (7); *S. paradoxus* CBS 2908 cybrid (8) parent (9); *S. paradoxus* CBS 7400 cybrid (10) parent (11); *S. paradoxus* CBS 432 cybrid (12) parent (13); size standard λ/PstI (14). **(B)** *Hinf*I digest of genomic DNA separated on 1% agarose gel. Lanes: *S. bayanus* CBS 380 cybrids (1–6) parent (7); size standard λ/PstI (8). **(C)** *EcoR*V digest of mtDNA separated on 1% agarose gel. Lanes: size standard λ/PstI (1, 14); *S. kudriavzevii* CBS 8840 cybrid (2) parent (3); *S. mikatae* CBS 8839 cybrid (4) parent (5); *S. cariocanus* CBS 7994 cybrid (6) parent (7); *S. paradoxus* CBS 2908 cybrid (8) parent (9); *S. paradoxus* CBS 7400 cybrid (10) parent (11); *S. paradoxus* CBS 432 cybrid (12) parent (13); size standard λ/PstI (14). **(D)** Pulse-field electrophoresis of cybrids (1–8) and parental species (9–16): Lanes 1–9 *S. cerevisiae* cybrids with mt genomes from *S. bayanus* CBS 380 (1); *S. mikatae* CBS 8839 (2); *S. paradoxus* CBS 7400 (3); *S. paradoxus* CBS 2908 (4); *S. kudriavzevii* CBS 8840 (5); *S. cariocanus* CBS 7994 (6); *S. paradoxus* CBS 432 (7); *S. cerevisiae* strain W303 1A (8); MCC109 ρ^0^ parental strain (9); *S. paradoxus* CBS 432 (10); *S. cariocanus* CBS 7994 (11); *S. paradoxus* CBS 2908 (12); *S. kudriavzevii* CBS 8840 (13); *S. mikatae* CBS 8839 (14) *S. bayanus* CBS 380 (15); *S. cerevisiae* strain W303 1A (16).

The presence and origin of mitochondrial genomes can be unambiguously assigned according to restriction fragment polymorphisms that have long been used for molecular “typing” of different strain-to-strain variations in yeasts (e.g., Querol et al., 1992; Špírek et al., 2000). Consequently *Hin*fI or *EcoR*V restriction profiles of all the cybrids chosen for further study were identical to the mtDNA profiles from parent species, which excludes the rearrangement of DNA (Figures 2A–C). The authenticity of transferred mt genomes was confirmed also by whole genome sequencing. The very rare event of single chromosome transfer is known if FOA selection pressure is applied (Nilsson-Tillgren et al., 1980), and this could possibly be responsible for compatibility. Cybrid karyotypes were not distinguishable from the original respiration deficient *S. cerevisiae* strain but variable enough to distinguish hybrids with both genomes and some of single chromosome transfers (Figure 2D; Marinoni et al., 1999). The uniform karyotype excludes the heterokaroyn variants and the transmission of intact nuclei from the mitochondrial donors. Auxotrophic phenotypes and mating type were well-preserved in all examined cybrids.

### The re-establishment of nucleo-mitochondrial compatibility with foreign mtDNA in *S. cerevisiae* often accompanies the adaptation process

**Figure 3:**
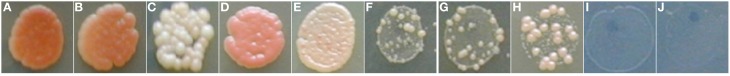
**Adaptation of cybrids.** Cells from single cybrid colony were diluted, spotted on the plates with the non-fermentable carbon source (YPGE) and cultivated for 7 days at 28°C. *S. cerevisiae* cybrids with mtDNA from: **(A)** *S. cerevisiae* W303 1A; **(B)** *S. cerevisiae* CBS 400; **(C)** *S. paradoxus* CBS 432; **(D)** *S. cariocanus* CBS 7994; **(E)** *S. kudriavzevii* CBS 8840; **(F)** *S. paradoxus* CBS 2908; **(G)** *S. paradoxus* CBS 7400; **(H)** *S. mikatae* CBS 8839; **(I)** *S. bayanus* CBS 380; **(J)** *S. cerevisiae* MCC109 ρ^0^.

Cybrids with partially re-established mitochondrial functions can be obtained on selection media containing glucose. If cells from a single cybrid colony are transferred from selection media (with glucose) on the plates with a non-fermentable carbon source such as glycerol and ethanol (YPGE), unambiguously good growth is displayed only in cybrids containing mitochondrial genome from various *S. cerevisiae* strains, the *S. paradoxus* strain CBS 432, and the *S. cariocanus* strain CBS 7994 (Figure 3, Table 1). Other cybrids gave rise to a mixed population that consisted mainly of poorly growing colonies. However, most of these cybrids with mtDNA from *S. paradoxus* CBS 7400, *S. paradoxus* CBS 2908, and *S. mikatae* CBS 8839 were capable of forming colonies on non-fermentable substrate after prolonged incubation on YPGE (Figure 3, Table 1), indicating an adaptation process. Cybrids with mt genome *S. kudriavzevii* CBS 8840 growing on glycerol also appeared, with a shorter but significant delay. Even after 2 months of incubation we did not observe any colony growing on glycerol among the cybrids containing mtDNA from the less-related yeasts of the *S. bayanus – uvarum* group.

**Table 1:** **Re-established growth on non-fermentable carbon source (YPGE)**.

***S. cerevisiae* cybrids (MCC109 ρ^0^) with mitochondrial genome from:**	**Proportion of colonies growing immediately on YPGE in (%)^b^**	**A period required for re-established growth on YPGE in (days)^c^**	**A period required for re-established growth on YPGE in (days) after second cytoduction to W303 1A strain^d^**
*S. cerevisae* W303 1A	100	0	0
*S. cerevisae* CBS 400^a^	100	0	0
*S. paradoxus* CBS 432	100	0	0
*S. paradoxus* CBS 2908	2	6–14	4
*S. paradoxus* CBS 7400	2	6–14	4
*S. kudriavzevii* CBS 8840	50	1–3	1
*S. mikatae* CBS 8839	15	6–14	4
*S. cariocanus* CBS 7994	100	0	0
*S. bayanus* CBS 380	0	>60	–
*S. cerevisae* MCC109 ρ^0^	0	>60	–

a The same was observed for other S. cerevisiae wild type strains listed in the Section Material and Methods and any examined respiring laboratory strain.

b Up to 50 colonies were examined.

c As visible colonies that arose from patch.

d Mitochondria transferred from primary cybrids to the strain W303 1A ρ^0^ transformed with the plasmid YEp352 by kar cross.

To understand the nature of the adaptation process, we transferred the cytoplasm from adapted cybrids capable of growing on YPGE again by cytoduction to a different *S. cerevisiae* W303 1A ρ^0^ strain (carrying YEp352 plasmid with *URA3* selection marker) in a ratio of 100:1. Putative cytoductant colonies were selected on glucose plates lacking uracil, and the presence of mtDNA was determined by DAPI staining. Colonies with W303 1A auxotrophic markers containing mtDNA were then transferred onto rich YPGE plates, and their growing ability was examined (Table 1). Again, the ability to utilize a non-fermentable carbon source in these second generation cybrids mimicked the behavior of cybrids obtained by first transfer from parental non-cerevisiae species. In spite of the reduced adaptation period, the proportionality confirms that transfer of nuclear genes from original parental species is not required.

**Figure 4:**
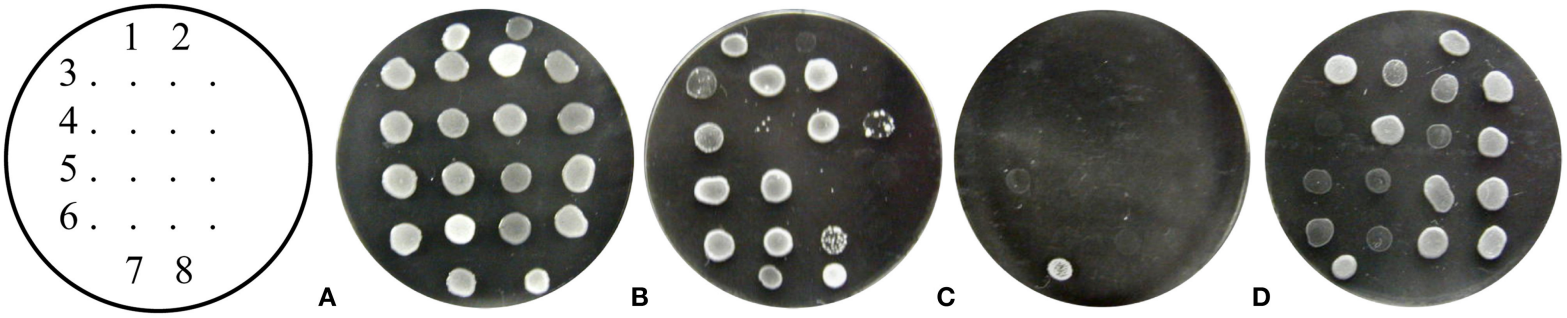
**Adaptive gain-of-mutation is linked to nuclear gene.** Zygotes from mating of adapted cybrids with *S. paradoxus* CBS 7400 mtDNA and W303 1A/YEp352 ρ^0^ strain were sporulated and tetrads dissected. Colonies from individual spores were spotted on different media. (3–6) Four individual tetrads; Controls: (1) MCC109 ρ^0^; (2) cybrid with S. *paradoxus* CBS 7400 mtDNA; (7) W303 1A ρ^+^; **(8)** W303 1A ρ^0^. Plates **(A)** YPD 30°C; **(B)** YPD 37°C; **(C)** YPGE 37°C; **(D)** YPGE 30°C. Cultivated 3 days at 28°C.

Reduced spore viability, used in taxonomic classification, is a very profound feature of interspecific yeast hybrids as well the landmark of nucleo-mitochondrial Dobzhansky–Muller incompatibility (Naumov et al., 2000; Chou and Leu, 2010). Therefore, *S. cerevisiae* cybrids repopulated with foreign mtDNA and capable of growing on a non-fermentable carbon source (adapted) were crossed with the *S. cerevisiae* W303 1A ρ^0^ strain carrying the YEp352 plasmid. Diploids were selected on the minimal media supplemented with adenine, and their sporulation ability was examined. All diploids could sporulate and the majority of spores (from 70 to 90%) germinated, suggesting the absence of Dobzhansky–Muller incompatibility pairs. In addition, spores from asci were separated by micromanipulator and full tetrads were tested for auxotrophic requirements and the ability to grow on YPGE. The segregation of the ability to grow on glycerol–ethanol when the mating partner was cybrid harboring mtDNA from *S. paradoxus* CBS 432, *S. cariocanus* CBS 7994, or any *S. cerevisiae* was at a ratio of 4:0, which emphasizes the direct compatibility of mitochondrial and nuclear genomes from different species. However, if one of the mating partners was a cybrid with mtDNA from *S. paradoxus* CBS 2908, *S. paradoxus* CBS 7400, *S. mikatae* CBS 8839, or *S. cariocanus* CBS 7994, the ability to grow on glycerol and ethanol segregated at a 2:2 ratio (Figure 4), indicating a single gene gain-of-function, responsible for the adaptation phenomenon. In the case of cybrids with *S. kudriavzevii* CBS 8840 mtDNA, the ratio was unexpectedly 3:1, although a small number of tetrads segregated at a ratio of 2:2 or 4:0, suggesting most likely the gain-of-function in two different genes involved in the adaptation process. The authenticity of the tetrads was always confirmed by the segregation of nuclear markers.

### Effectiveness of oxidative phosphorylation in the xenomitochondrial cybrids

The performance of oxidative phosphorylation was assessed according to the rate of oxygen consumption, measured in cultures grown in liquid YPD medium followed by a 12 h cultivation in YPGE, which allows non-adapted variants to be compared as well. The respiration capacity of cybrids that display good nucleo-mitochondrial compatibility (compatible mt genomes) from the *S. paradoxus* strain CBS 432 and *S. cariocanus* CBS 7994 decreased to 40% of the *S. cerevisiae* (Table 2). The oxygen consumption was reduced to 20–30% in adapted cybrids with mtDNA from *S. kudriavzevii, S. paradoxus* CBS 7400, *S. paradoxus* CBS 2908, and *S. mikatae* CBS 8839. A threshold respiration rate about 2–10% of the wild type can be detected in non-adaptated variants but not in a cybrid with *S. bayanus* mtDNA.

**Table 2:** **Respiration capacity of cybrids**.

***S. cerevisiae* cybrids (MCC109 ρ^0^) with mitochondrial genome from:**	Respiration capacity (nmol O_2_/min/5 × 10^8^ cells)		Relative respiration capacity (%)	
	**A**	**NA**	**A**	**NA**
*S. cerevisiae* W303 1A	190 ± 50	–	100	–
*S. cerevisiae* CBS 400	180	–	95	–
*S. paradoxus* CBS 432	80	–	42	–
*S. cariocanus* CBS 7994	77	–	41	–
*S. kudriavzevii* CBS 8840	64.6	33	34	17
*S. paradoxus* CBS 7400	43.7	3.88	23	2
*S. paradoxus* CBS 2908	55	3.81	29	2
*S. mikatae* CBS 8839	64	4	34	2
*S. bayanus* CBS 380	–	0	–	0
*S. cerevisiae* MCC109 ρ^0^	–	0	–	0

A, adapted; NA, non-adapted.

**Figure 5:**
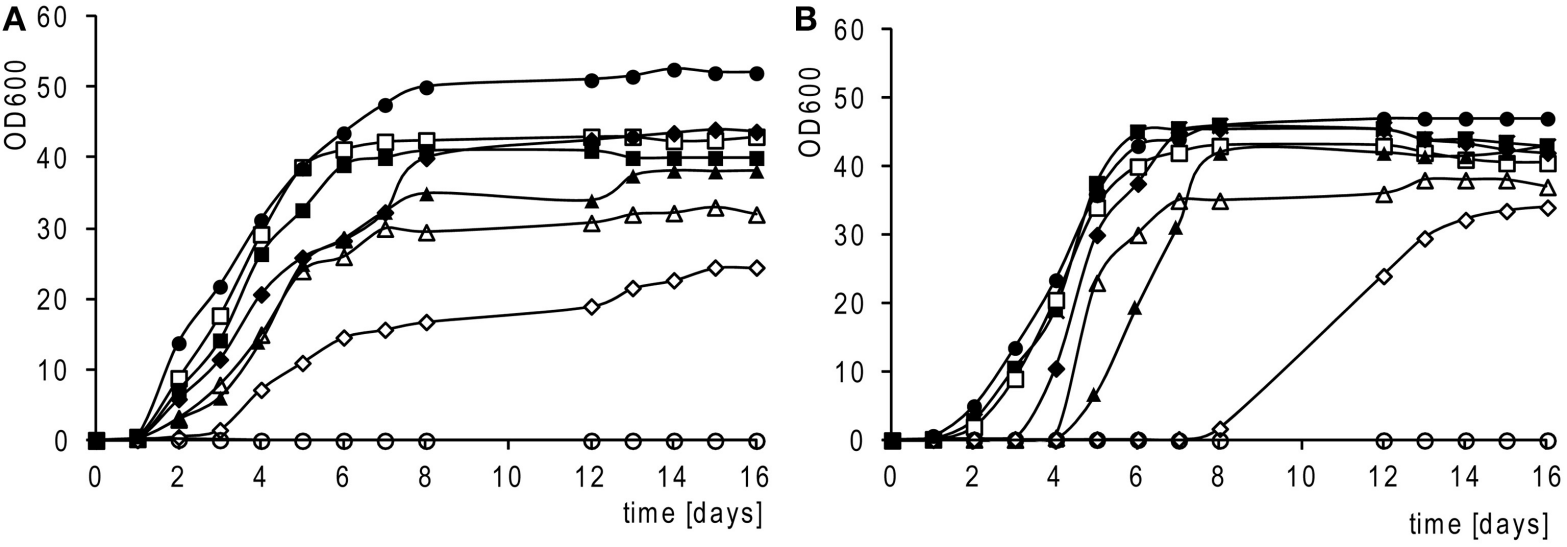
**Growth rates of adapted cybrids.** Cells grown in liquid complete medium with glycerol and ethanol (YPGE). **(A)** Inoculum from YPGE; **(B)** inoculum from YPD; *S. cerevisiae* cybrids (strain MCC109 ρ^0^) with mt genomes from: *S. cerevisiae* strain W303 1A (•); *S. bayanus* CBS 380 (°); *S. paradoxus* CBS 432 (■); *S. cariocanus* CBS 7994 (□); *S. paradoxus* CBS 7400 (▲); *S. paradoxus* CBS 7400; (Δ); *S. kudriavzevii* (CBS 8840) (♦); *S. mikatae* CBS 8839 (◊); 1 OD600 unit equals 6 × 10^7^ cells/ml.

The growth rate on the medium with non-fermentable carbon is widely used as a good criterion for assessing the phenotype of various mitochondrial mutations. Figure 5A shows that cybrids with mitochondria from the *S. paradoxus* strain CBS 432 and *S. cariocanus* CBS 7994 grow nearly at the same rate as cybrids with *S. cerevisiae* mitochondria. Cybrids requiring an adaptation period (*S. paradoxus* CBS 7400, *S. paradoxus* CBS 2908, *S. mikatae* CBS 8839) exhibit a significantly slower growth rate. Besides the slower growth rate, they exhibit an extremely long lag phase (3–8 days) if they are transferred from glucose to glycerol (Figure 5B).

### Adaptation introduces temperature sensitive phenotype on glucose

**Figure 6:**
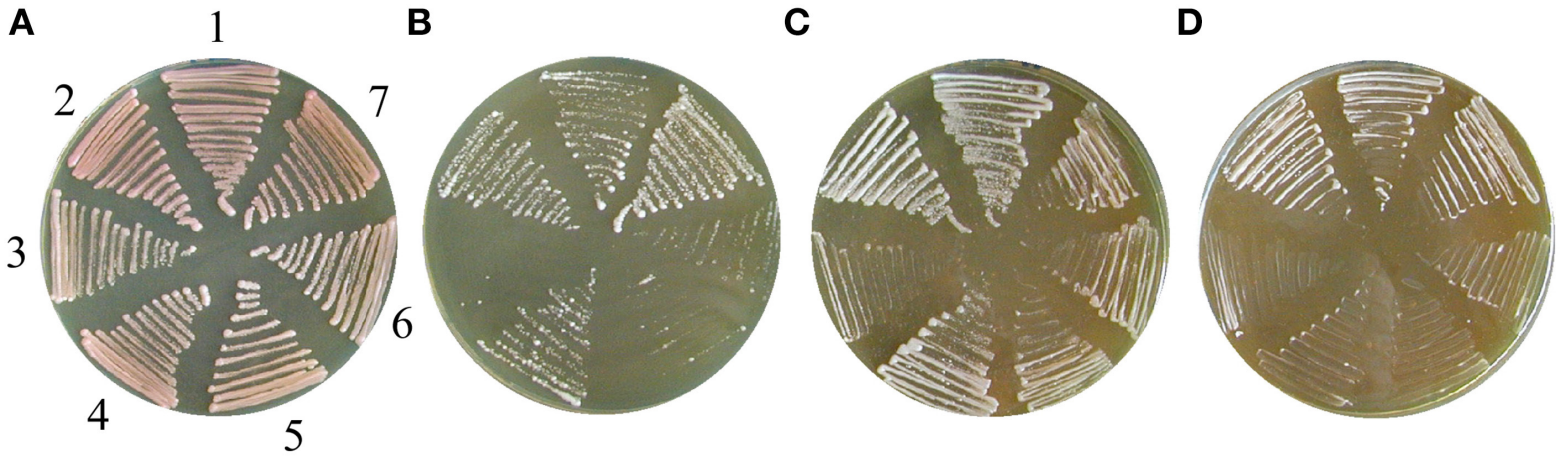
**Temperature sensitive growth of xenomitochondrial cybrids.** Cultivation **(A)** YPD 30°C, **(B)** YPD 37°C **(C)** YPGE 30°C **(D)** YPGE 37°C: *S. cerevisiae* cybrids with mitochondrial genomes from *S. cerevisiae* strain W303 1A (1); *S. paradoxus* CBS 432 (2); *S. paradoxus* CBS 2908 (3); *S. kudriavzevii* CBS 8840 (4); *S. mikatae* CBS 8839 (5); *S. paradoxus* CBS 7400 (6); *S. cariocanus* CBS 7994 (7).

To shed more light on the scale of the communication ability of xenomitochondrial cybrids, we examined their ability to grow at an elevated temperature. While cybrids with mtDNA from *S. cerevisiae* and compatible *Saccharomyces* strains (*S. paradoxus* CBS 432 and *S. cariocanus* CBS 7994) were capable of growing at 37°C, even on non-fermentable carbon sources, other cybrids with non-cerevisiae mtDNA were not. Surprisingly, they were not capable of growing at an elevated temperature even on YPD with glucose as a carbon source, while the parental *S. cerevisiae* ρ^0^ mutant was. Their growth was abolished even after the transfer from 37 to 30°C, indicating the lethal effect of elevated temperature (Figure 6).

### Mitochondrial *cox1* gene is the main determinant of xenonucleo-mitochondrial compatibility

**Figure 7:**
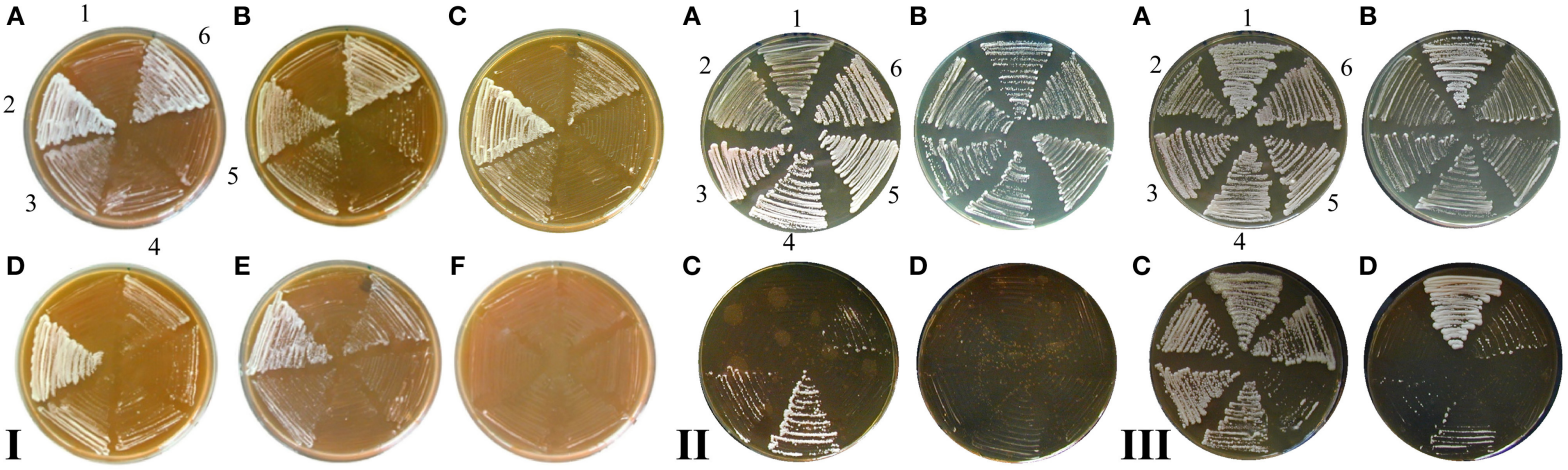
***Cox1* gene as the main determinant of xenonucleo-mitochondrial (in)compatibility. (I)** Growth of non-adapted *S. cerevisiae* cybrids with mitochondrial genomes from *S. paradoxus* CBS 7400 **(A)**; *S. paradoxus* CBS 2908 **(B)**; *S. mikatae* CBS 8839 **(C)**; *S. kudriavzevii* CBS 8840 **(D)**; *S. bayanus* CBS 380 **(E)**; MCC109 ρ^0^ Strain (negative control) **(F);** after mating to different *mit*^−^ strains: (1) MD79 (*mit*^−^ in *cox*1), (2) akar170 (*mit*^−^ in *trn*Asp), (3) M1301 (*mit*^−^ in *cob*), (4) AD1 (*mit*^−^ in *cox*1); (5) 5B (*mit*^−^ in *cox*1); (6) K2145 (*mit*^−^ in *cox*2). Cultivated on YPGE plates for 4 days at 30°C. Growth of non-adapted cybrids after the mating with *S. cerevisiae* DBY 747 ρ^0^ **(II)** and *S. cerevisiae* DBY 747 ρ^−^ containing intronless *cox1* gene **(III)** mit^−^ strain 2612 (positive control) (1); *S. cerevisiae* cybrids with mitochondrial genomes from: *S. paradoxus* CBS 7400 (2); *S. paradoxus* CBS 2908 (3); *S. kudriavzevii* CBS 8840 (4); *S. mikatae* CBS 8839 (5); *S. paradoxus* CBS 7400 (6). Plates **(A)** YPD 30°C, **(B)** YPD 37°C, **(C)** YPGE 30°C, **(D)** YPGE 37°C. Cultivation 4 days.

The segment of mtDNA responsible for interspecific incompatibility can be determined by petite mapping (Fox et al., 1991). Therefore, we first crossed all non-adapted cybrids with *S. cerevisiae mit*^−^ strains carrying mutations in single mitochondrial genes *cox1*, *trnD*, *cob*, and *cox2* (details listed in the section Materials and Methods). All combinations grew well on YPGE plates, but not diploids from the crosses of three different *cox1* mutants (carrying deletion of exon2 AD1, 5B or 8.2 kb deletion of exon2–intron I5β MD 79) suggested an impaired compatibility associated with the *cox1* gene (Figure 7). This outcome was confirmed by petite mapping using ρ^−^ mutants prepared from the *S. cerevisiae* DBY 747 strain harboring intronless mtDNA (Seraphin et al., 1987). All ρ^−^ strains able to complement the growth defects of non-adapted cybrids carried mtDNA fragments containing the entire *cox1* gene, which was confirmed by genetic and physical mapping.

**Figure 8:**
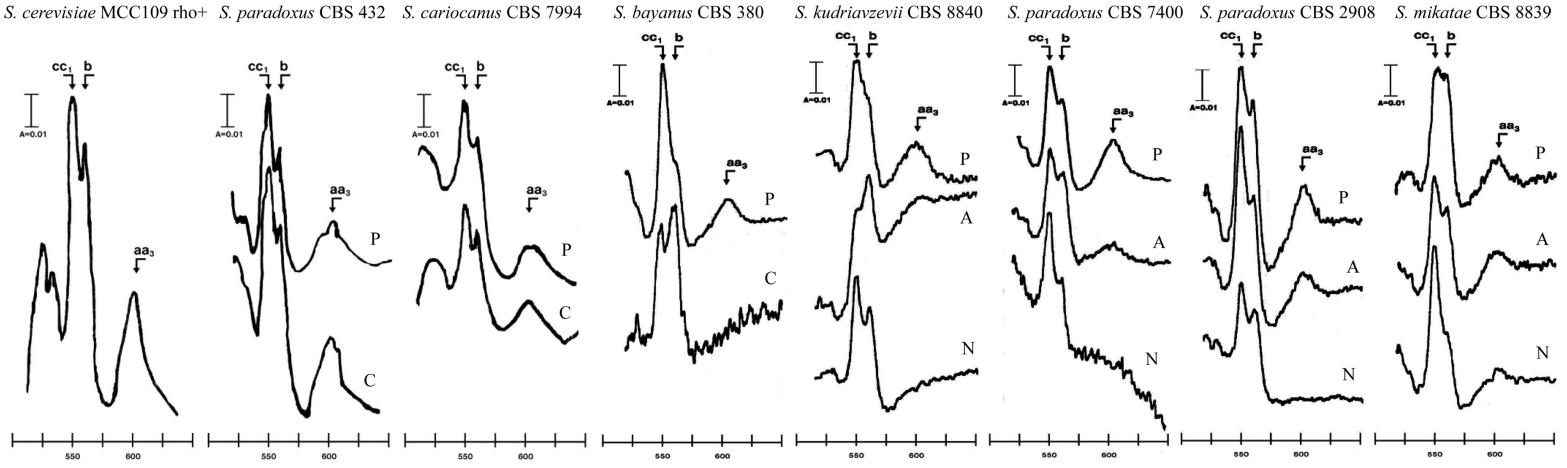
**Cytochrome spectra of parental species and xenomitochondrial cybrids.** Absorption maxima for cytochrome *aa*_3_ – 603 nm; cytochrome *b – 560 nm;* cytochrome *cc1* – 550 nm. Abbreviations: P, parent; C, cybrid; A, adapted cybrid; N, non-adapted cybrid.

A defect in the large subunit of the *cox1* gene confirmed cytochrome spectra of reduced vs. oxidized mitochondrial extracts (Tzagoloff et al., 1975). The ratio of cytochromes *cc1*-*b*-*aa3* similar to the wild type is maintained in *S. cerevisiae* cybrids, with *S. cariocanus* and *S. paradoxus* CBS 432 mtDNA. In other cybrids with only partially compatible genomes, a cytochrome *aa3* peak is barely detectable in non-adapted forms and reaches about 50% of the wild level after adaptation. In *S. cerevisiae* with *S. bayanus* mtDNA the cytochrome *aa3* signal is missing. It appears that the defect in *cox1* expression is a major drawback in the interspecific nucleo-mitochondrial (in)compatibility in *Saccharomyces* (Figure 8).

### The ability of *cox1*-I3β intron to splice in a new environment is the main determinant of xenonucleo-mitochondrial compatibility

**Figure 9:**
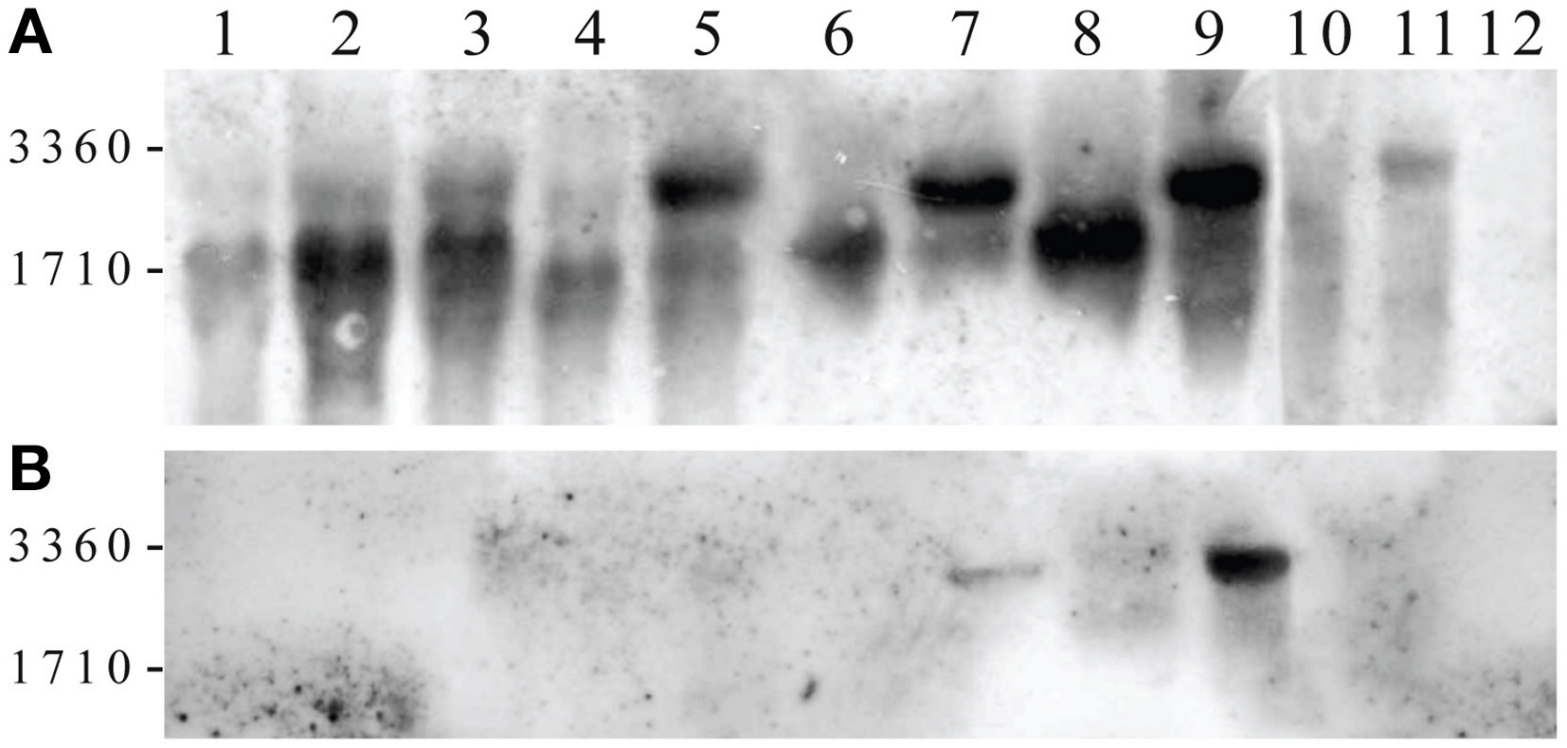
**Northern blot of RNA from parental species and adapted cybrids.** RNA separated on agarose–formaldehyde gel, transferred on nylon membrane was hybridized with the probe specific to first two exons from *S. cerevisiae cox1* gene. **(A)** Lanes: *S. cerevisiae* (W303 1A ρ^+^) (1); *S. paradoxus* CBS 432 parent (2), cybrid (3); *S. kudriavzevii* CBS 8840 parent (4), cybrid (5); *S. paradoxus* CBS 2908 parent (6), cybrid (7); *S. paradoxus* CBS 7400 parent (8), cybrid (9), *S. mikatae* CBS 8839 parent (10), cybrid (11); *S. cerevisiae* MCC109 ρ^0^ (12). **(B)** The same membrane stripped and hybridized with probe specific to I3β from *S. paradoxus* CBS 7400. Markers: 3360 nt 26S rRNA; 1710 nt 18S rRNA.

The inability of the *cox1*-I3β intron to splice in a new environment has been already suspected to be the main obstacle to *S. cerevisiae/S. paradoxus* interspecific nucleo-mitochondrial communication (Kotylak et al., 1985; Tian et al., 1993) due to the divergence of the Mrs1p splicing factor (Herbert et al., 1992; Chou et al., 2010). Therefore, we amplified the region of the insertion in all species involved in our study. *Cox1*-I3β in different sizes was found in any non-cerevisiae *Saccharomyces*, but never in the 20 examined *Saccharomyces* strains isolated from different geographical regions (not shown). In order to understand its role in the adaptation process, whole-cell RNA from adapted cybrids was analyzed by Northern blot. Hybridization with the probe specific to the first two *S. cerevisiae cox1* exons revealed two different bands in which one was linked to spliced *cox1* mRNA about 1700 nt long, and the second was linked to longer mRNA 2500 nt in size, corresponding to the *cox1* premRNA with unspliced I3β intron RNA (Figure 9A). The signal intensity of longer form was very profound in cybrids, but was noticeable in parental strains only after overexposure. The membrane was stripped and hybridized with a probe specific to I3β from *S. paradoxus* CBS 7400 (Figure 9B). In this case, the probe hybridized only to the longer *cox1* mRNA, providing evidence that it contains an unspliced I3β intervening sequence. Apparently, the lower levels of *aa3* cytochromes are associated with a lower level of *cox1* mRNA. The effect is strain specific, because in the cybrid with *S. paradoxus* CBS 432 mtDNA, the I3β splicing efficiency is the same as in the parental strain (Figure 9A).

### Splicing defect can be suppressed by variety of nuclear factors (xenoadjustors)

**Figure 10:**
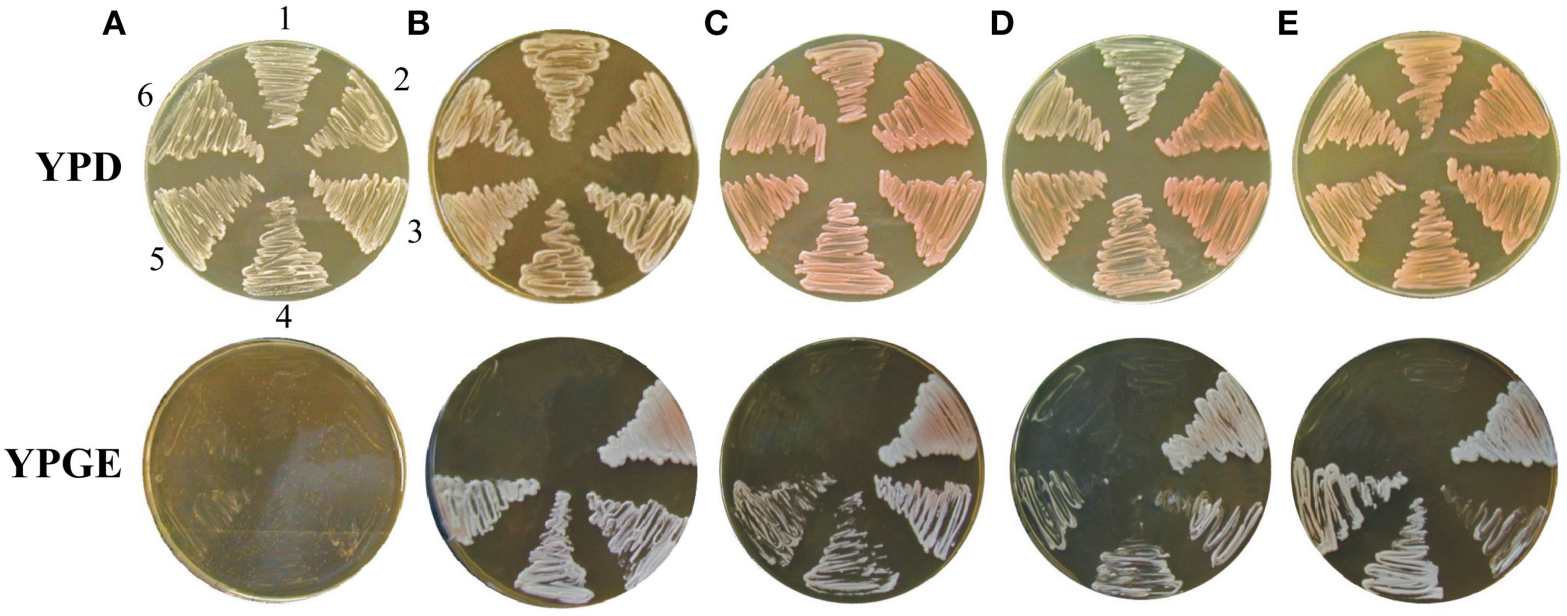
**The ability of non-adapted cybrids transformed with xenoadjustors to grow on non-fermentable carbon source.** *S. cerevisiae* cybrids with mitochondrial genomes from **(A)** *S. bayanus* CBS 380 **(B)** *S. paradoxus* CBS 2908 **(C)** *S. paradoxus* CBS 7400 **(D)** *S. mikatae* CBS 8839 **(E)** *S. kudriavzevii* CBS 8840 transformed with plasmid containing genes: 1 – none (pYES2), 2 – *MRP13*, 3 – *MRS2*, 4 – *MRS3*, 5 – *MRS4*, 6 –none (YEP24). Cultivation 4 days.

Non-adapted cybrids provide a unique opportunity to screen for other fine Dobzhansky–Muller incompatibility pairs. Therefore, the *S. cerevisiae* strain carrying mtDNA from *S. paradoxus* CBS 7400 was transformed with two different libraries (centromeric in pYES plasmid with inserts cloned under the Gal promoter, Ramer et al., 1992; multicopy in pFL plasmid, Rose and Broach, 1991). About 40,000 colonies from both libraries were replica-plated on YPGE plates and those with plasmid-dependent YPGE growth were selected. From the low copy pYES library we obtained 13 clones, all with the insert of the *MRP13* (*mitochondrial ribosomal protein*) gene involved in mitochondrial protein synthesis (Partaledis and Mason, 1988) inserted in both directions. In the case of the multicopy pFL library, two clones carried the *MRS3* and 3 the *MRS4 (mitochondrial RNA splicing factors)* gene belonging to the group of unspecific mitochondrial splicing factors (Wiesenberger et al., 1991). Therefore, we also examined the other family member *MRS2* on multicopy plasmid that supported the growth on YPGE plates. All four genes were universally capable of suppressing the glycerol growth defect in any of the non-adapted xenomitochondrial cybrids, except those containing *S. bayanus* mtDNA. However, all these “adjustors” were active only at 28°C but not at 37°C (Figure 10). The role of *MRS* genes is obvious as they have been isolated as suppressors of mitochondrial splicing mutants (Koll et al., 1987). While *MRS* genes on multicopy plasmids introduced into the DBY 747/M1301 strain, carrying a mutation in the first intron of *cob* gene (*cob bI1M1301*), were able to suppress the glycerol growth defect, *MRP13* was not (not shown). Apparently, the suppressor effect of this gene is associated with a different process. To shed light on the role of *MRP13* we disrupted the gene in an adapted cybrid with mtDNA from *S. paradoxus* CBS 7400 as well as in *S. cerevisiae* parental strain W303 1A. While *MRP13* disruption in the W303 1A *S. cerevisiae* laboratory strain did not exhibit any apparent phenotype, the adapted variant of xenomitochondrial cybrid lost the ability to grow on glycerol, suggesting the role of this gene in the natural adaptation process. When this strain was crossed with W303 1B ρ^0^, the ability to grow on glycerol was segregated at a 0:4 ratio, which confirms the role of this gene. After prolonged growth for 7–14 days by all patches with the disrupted *MRP13* gene on YPGE, isolated colonies appeared that indicated the ability of cells to substitute for the knocking out of *MRP13.* Apparently, the impaired interspecific nucleo-mitochondrial communication can be compensated for by gain of function that involves multiple genes.

## Discussion

*S. cerevisiae* xenomitochondrial cybrids can be prepared simply by the mating of ρ^0^ strains with impaired karyogamy and germinating spores of different *Saccharomyces* taking advantage of the *ura3* mutation and fluororotic acid selection. Cytoduction allows the construction of cybrids that grow poorly if at all on a non-fermentable carbon source due to their ability to survive the prolonged incubation on solid synthetic media with FOA better.

In general, all *S. cerevisiae* xenomitochondrial cybrids fall into three categories. The first includes cybrids containing mitochondrial genome from other *S. cerevisiae* strains, and two different species *S. paradoxus* CBS 432 and *S. cariocanus* CBS 7994 (Table 2). Besides a decreased respiration rate, the last two cases do not exhibit any stronger phenotype. They are metabolically and genetically similar to cybrids with mitochondrial genomes from different *S. cerevisiae* strains. Their growth rate, cytochrome content, and respiration rate is slightly lower, and they also display a short lag phase extension after the shift from glucose to glycerol-ethanol media. Cytoduction experiments and the segregation of glycerol grown phenotype in a 4:0 ratio in tetrads after mating to *S. cerevisiae* ρ^0^ confirmed that these two mtDNA do not require changes in any nuclear gene to re-establish usable oxidative phosphorylation. Mismatched mitochondrial and nuclear genomes from two different *S. cerevisiae* strains often result in very profound reduced fitness on non-fermentable medium (Zeyl et al., 2005; Dimitrov et al., 2009; Paliwal et al., 2014). It is difficult to decide whether minor incompatibilities in this class of compatible cybrids are due to nucleo-mitochondrial epistasis or whether some “interspecific” factor may play a role.

The second class consists of cybrids containing mtDNA from two other *S. paradoxus* strains, *S. mikatae* and *S. kudriavzevii*, which exhibit an impaired performance of oxidative phosphorylation and a poor ability to grow on non-fermentable substrate after the transplacement of mitochondria. However, long-term incubation on glycerol media that may last for several weeks initiates the growth of adapted variants. Their respiration capacity is as low as 2–3% and increases to 20–30% of wild type level due to the elevated cytochrome *aa3* content resulting from improved *cox1*-I3β intron splicing. This period of adaptation feature has not been observed in any other well-characterized xenomitochondrial cybrids from primate or rodent cells, and it seems to be yeast specific (Dey et al., 2000; McKenzie and Trounce, 2000; Yamaoka et al., 2000; Burton et al., 2013). The capability of growing on glycerol is temperature sensitive and this gain-of-function is associated with the loss of the ability to grow at 37°C even on glucose, a fermentable substrate that can be utilized anaerobically. With the exception of cybrid with *S. kudriavzevii* and *S. paradoxus* CBS 432 mtDNA, the exposure to an elevated temperature of 37°C is lethal and cells do not grow after replica-plating on a fresh YPD plate. They also exhibit a slower growth rate on glycerol, and a very remarkable feature is the extremely long lag phase after the shift from glucose to glycerol–ethanol media (Figure 5B). They appear to behave like nucleo-mitochondrial communication mutants. We failed to find any similar phenotype from the literature search, but temperature sensitive mutations in general indicate defects in the protein-coding genes often essential for cell viability (Hampsey, 1997). Conditionally, lethal growth on a non-fermentable carbon source is quite frequent and has been reported as a consequence of deletion/mutation in a few genes, for example, *ABF2* coding for HMG-like DNA binding protein (Kao et al., 1993) and *MMF1* for protein with unknown function, but present in eukaryotes and prokaryotes (Oxelmark et al., 2000); however, these mutants are capable of growing at an elevated temperature on glucose. The respiration capacity of adapted cybrids is sufficient to perform a normal yeast cell cycle. They can sporulate after mating to *S. cerevisiae* ρ^0^ strains and tetrad analysis has demonstrated their ability to grow on glycerol segregated mostly at a 2:2 ratio. The most plausible explanation for this segregation is some unspecified adaptive gain-of-mutation(s). After prolonged cultivation, single colonies arose from the patches of originally non-growing spores on glycerol media, which underlines their ability to adapt under selection pressure. A similar “adaptation” phenotype has been reported for mutants lacking mitochondrial porin. Deletion of the gene impairs some respiratory functions and therefore the growth on non-fermentable carbon sources; however, after a lag phase porinless mutant cells adapt to growth on glycerol (Dihanich et al., 1987). Apparently, this type behavior is not exceptional if the introduction of foreign mtDNA into *S. cerevisiae* is considered as the mutation. In yeast cells, such intervention puts pressure on the organism's genome to compensate for it so that it leads to a mutation in other genes (Teng et al., 2013).

**Figure 11:**
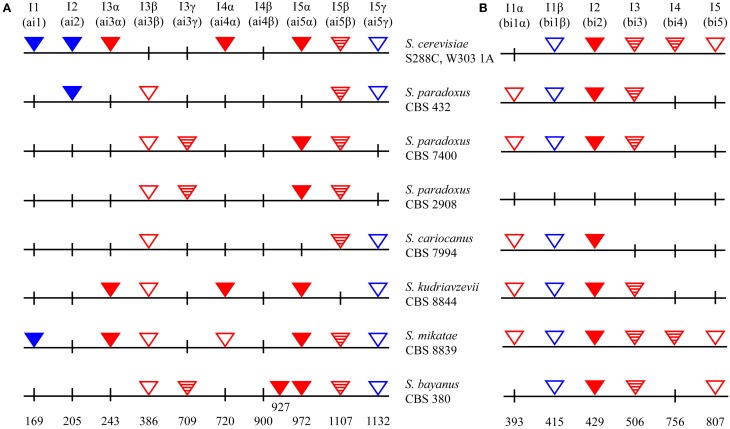
**Intron insertion sites in *Saccharomyces* mtDNA (A) *cox1;* (B) *cob* genes.** Red – Group I introns; Blue – Group II introns; filled – with homing endonuclease (known as mobile in *S. cerevisiae*); checkered – with ORF (not known as mobile in *S. cerevisiae*); empty triangles – ORF absent. *S. cerevisiae* mtDNA according to Foury et al. (1998).

Northern blot revealed the limited ability of *cox1*-I3β to splice as the most plausible explanation of the different behavior observed in these two cybrid classes. We made much effort to obtain the clean elimination of *cox1*-I3β by cultivation of cybrids at 37°C. This should have introduced selection pressure that resembles intron mutation, principally used in the preparation of intronless mtDNA strain (Seraphin et al., 1987). However, *cox1*-I3β intronless variants were not obtained in spite the fact that two of transferred genomes carried group II introns needed for *in vivo* deletion of mitochondrial introns (Figure 11; Levra-Juillet et al., 1989). Also, we tried to eliminate the intron by mating with appropriate petites, but several tens of petite strains capable of complementing the glycerol growth defect harbored a much longer *cox1* segment. Cytoduction experiments showed that the splicing factor responsible for *cox1*-I3β splicing in the *S. paradoxus* strain CBS 432 and *S. cariocanus* CBS 7994 mt genomes is coded by mtDNA. To find the particular gene we made a collection of petite mutants from a cybrid with *S. paradoxus* CBS 432 mtDNA and crossed them with the non-adapted cybrid with mtDNA from *S. paradoxus* CBS 7400. All petite genomes capable of complementing the glycerol growth defect were mapped to the *cox1* gene. To shed more light on this puzzling difference, we sequenced *cox1* (and entire mtDNAs) in all non-saccharomyces species as well in cybrids used in this study (Figure 11). The divergence in the *cox1*-I3β sequence cannot by itself explain the differences in splicing efficiency among the different *S. paradoxus* strains. Sequencing revealed only minor changes in the loop in P8 (insertion of ATAATAA pos. 298 and (C→A) transition in pos. 328, out of the catalytic core of intron) between the form in *S. paradoxus* CBS 432 (does not need adaptation) and two other *S. paradoxus* strains (needing adaptation; Prochazka et al., 2012, Supplementary Material). The comparison of open reading frames, hypothetically coded for maturases, directed our attention toward the C terminus of the open reading frame inside *cox1*-I5β, which is not fused to the upstream exon. It is nearly identical in mtDNAs of *S. paradoxus* strain CBS 432 and *S. cariocanus* CBS 7994, but interrupted by GC cluster in two strains that require an adaptation period. However, *cox1*-I5β is not mobile and its splicing requires at least five different nuclei-coded splicing factors (Mrs1, Mss116, Pet54, and perhaps open reading frame coded by *cob*-I3; Watts et al., 2011).

We tried to identify a fast evolving nuclear gene allowing the adaptation to “foreign” mtDNAs. A remarkable (tax reminiscent) feature of the adaptation process was the loss of the ability to grow on glucose at elevated temperature. The phenotype was maintained even after mtDNA elimination, and was recessive as diploids from crosses with the *S. cerevisiae* ρ^0^ strain were temperature resistant. Therefore, we attempted to find a gene from low copy and multicopy libraries by selecting for the ability to grow at 37°C, but we did not succeed, mainly due to high rate of reversion accompanied by the loss of the ability to grow well on glycerol.

When non-adapted cybrid with mtDNA *S. paradoxus* CBS 7400 was transformed with libraries and glycerol growing colonies, selected *MRS2, MRS3, MRS*4 genes were identified as multicopy suppressors. All these genes were originally isolated as suppressors of a mutation in the first introns of *cob* and *cox1* genes in *S. cerevisiae* impaired in splicing (Koll et al., 1987; Waldherr et al., 1993). All *MRSs* code for mitochondrial transport proteins (reviewed by Arco and Satrústegui, 2005). Mrs2p was identified as a Mg^2+^ carrier (Kolisek et al., 2003) and another two carriers, Mrs3p and Mrs4p, were shown to be involved in iron transport (Foury and Roganti, 2002; Mühlenhoff et al., 2003). Apparently, none of these genes is responsible for adaptation. Overexpression of the *MRS3* and *MRS4* causes temperature-dependent growth on glycerol but not on glucose, and the disruption of these genes affected neither the mitochondrial functions nor cellular viability (Wiesenberger et al., 1991). Strains with *MRS2* disruption are unable to grow on non-fermentable substrates (Wiesenberger et al., 1992). None of the *MRS* genes induce a temperature sensitive phenotype on glucose in non-adapted cybrids when overexpressed. *MRP13* belongs to different class of xenoadjustors. Like *MRS* genes, it is involved in ion trafficking (Eide et al., 2005), but it has been originally reported as a 35 kDa protein participating in mitochondrial protein synthesis as a structural component of the mitochondrial ribosome (Partaledis and Mason, 1988). It may be involved in premRNA intron splicing (Pandit et al., 2009), but it is one of the most regulated proteins, dependent on the state of the mitochondria. Like *CIT2*, the level of the *MRP13* transcript is elevated twice in ρ^0^ cells (Traven et al., 2001). Despite high levels of the *MRP13* transcripts, protein did not accumulate, suggesting that the protein is relatively unstable in the absence of ribosome assembly. In ρ^+^ cells, transcript levels were repressed several fold when on glucose compared to a non-fermentable carbon source (Partaledis and Mason, 1988). In contrast to *MRS* genes, *MRP13* on low copy plasmid is unable to facilitate the splicing of mitochondrial intron mutants and therefore it should affect the *cox1* expression in a different way. It is a much better candidate for a natural adaptation gene because its disruption/deletion abolishes the ability to grow on glycerol in xenomitochondrial cybrids, but not in cells containing aboriginal *S. cerevisiae* mtDNA. However, its overexpression does not trigger the temperature sensitive phenotype on glucose.

**Figure 12:**
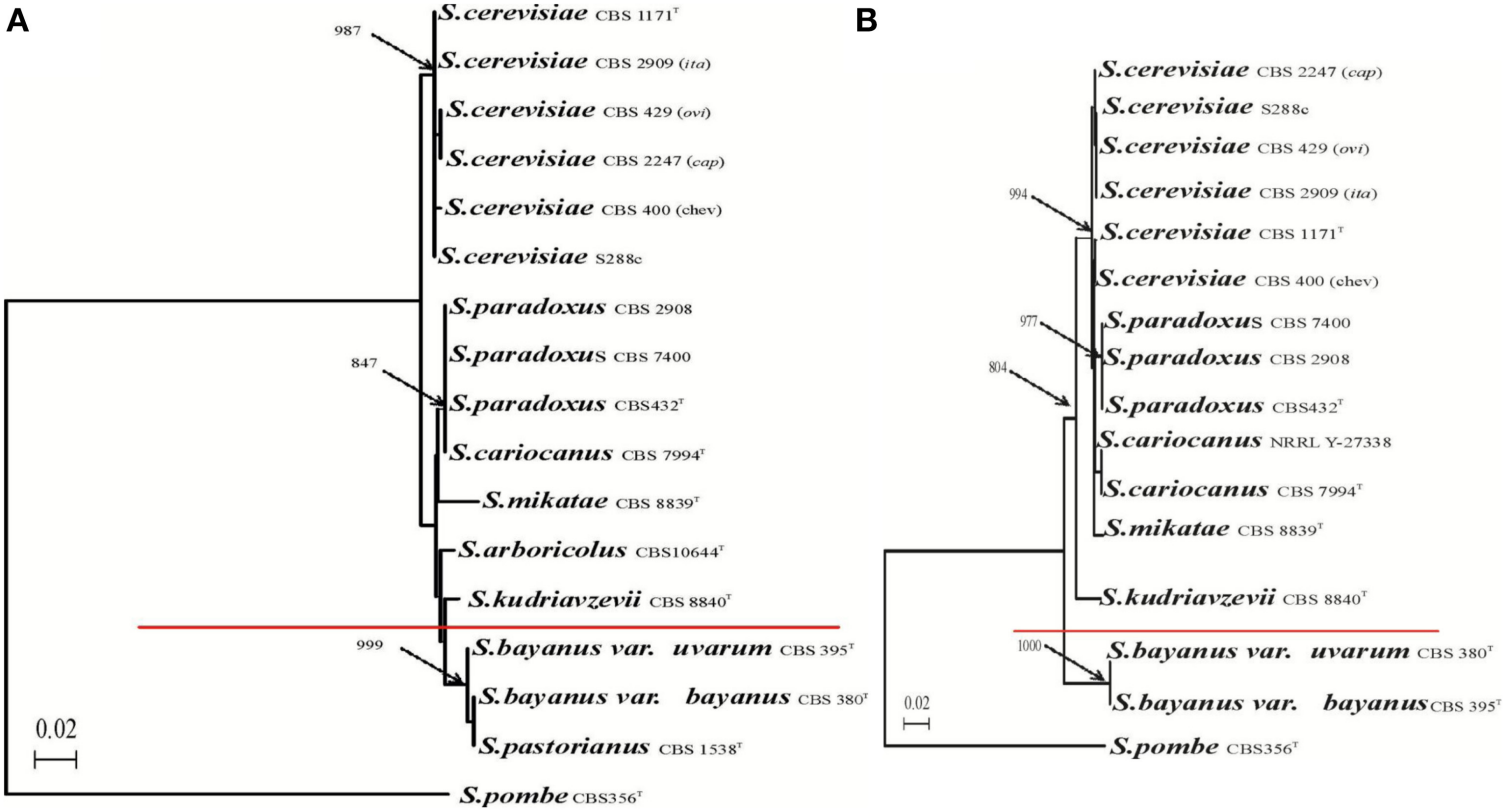
**Compatibility limit of *S. cerevisiae* nuclear genome and mitochondrial genomes from related yeasts.** Phylogenetic tree of *Saccharomyces* species based on the comparison of **(A)** nuclear 26S rDNAs sequence and **(B)** mitochondrial *cox2* gene. Branch lengths, based on nucleotide substitutions, are indicated by the bar. Bootstrap values of 1000 replications are marked by arrows at the branches points. Values less than 50% are not given. *Schizosaccharomyces* pombe was used as outgroup.

The third class is the *S. cerevisiae* cybrid with mtDNA from *S. bayanus* CBS 380, which is unable to re-establish growth on the non-fermentable carbon source even after 60 days on YPGE plates. This cybrid does not respire and glycerol growth can be restored only after mating with *mit*^−^, which does not have a defect in the *cox1* gene. Respiring xenomitochondrial cybrids with *S. cerevisiae* mtDNA in *S. bayanus* cannot be prepared either (Sulo et al., 2003). This establishes the nucleo-mitochondrial compatibility limit of *S. cerevisiae* and other *Saccharomyces* species between *S. kudriavzevii* and *S. bayanus* (Figure 12). The numbers concerning the divergence of particular *Saccharomyces* species are still being developed. However, multiple calculations have estimated that *S. paradoxus* and *S. cerevisiae* diverged more than 7–10 million years ago and *S. cerevisiae, S. bayanus* more than 20 million years ago (www.genetics.wustl.edu/saccharomycesgenomes; Lin et al., 2006; Scannell et al., 2011). The *S. paradoxus/S. cerevisiae* rate of divergences compared with mammals (Dujon, 2006) corresponds to the human–rodents span and *S. cerevisiae/S. bayanus* to the human–birds distance. The limit confirms the transfer of *S. cerevisiae* mtDNA in the opposite direction. MtDNA containing all introns or intronless form is able to restore respiration in *S. paradoxus, S. kudriavzevii* and *S. mikatae* but not in *S. bayanus* to a level close to the original without adaptation period (unpublished). This emphasizes the unidirectional character of nucleo-mitochondrial incompatibilities and in particular the significant role of unusual *cox1*-I3β intron.

In conclusion, the interactions of *MRS1-cox1* considered as cytonuclear Dobzhansky–Muller pairs of speciation between *S. cerevisiae* and *S. paradoxus* are not strong enough to determine separation of species (Chou et al., 2010). Also, they do not play a dominant role in the separation between *S. cerevisiae* and the rest of the *Saccharomyces* species with the exception of species from the *S. bayanus*–*S. uvarum* clade (Figure 12). The compatibility is strain specific as the mitochondrial genomes can cooperate immediately and well-enough to support the utilization of non-fermentable substrate and sexual propagation. In the latter case, nuclear and mitochondrial genomes are capable of establishing collaborative communication after adaptation of one or several nuclear genes. Clear and strong cytonuclear incompatibility has been observed only between *S. cerevisiae* and *S. bayanus*, where additional Dobzhansky–Muller pairs have been reported (Chou et al., 2010).

Generally the post-zygotic barrier of speciation resulting in hybrid inviability and hybrid sterility has been attributed to a large number of genomic regions across a wide variety of species (for reviews Maheshwari and Barbash, 2011; Nosil and Schluter, 2011; Butlin et al., 2012; Mensch et al., 2013). In higher eukaryotes most of them involve chromosomal rearrangements and incompatibilities in nuclear Dobzhansky–Muller gene pairs (Maheshwari and Barbash, 2011). The yeasts with prevailing mito-nuclear incompatibilities are exception from this concept, although analogous mismatch have been found also in higher eukaryotes. MtDNA from chimpanzee, pigmy chimpanzee and gorilla but not from orangutan and less related primates is capable to restore oxidative phosphorylation in human cells devoid of mtDNA to essentially normal levels (Kenyon and Moraes, 1997; Barrientos et al., 1998). Similar relationships have been reported in mouse cybrids repopulated with mtDNA from various rodent species (Yamaoka et al., 2000; McKenzie et al., 2003, reviewed in Wolff et al., 2014). However, due to the uniparental inheritance of mitochondria, they are not considered as the main post-zygotic barrier. In yeast mitochondria are inherited biparentally that explains why cytonuclear incompatibility was preferentially observed in reproductive isolation (Lee et al., 2008; Chou et al., 2010). In addition, “yeast specific” combination of intron with species-specific splicing factor (such as incipient *MRS1-cox1*-I3β Dobzhansky–Muller pair) may play a significant role as post-zygotic barrier in other lower eukaryotes. Due to the horizontal transfer, mobile introns carrying homing endonuclease are able to “travel” between species in recurrent cycles of invasion, reading frame mutation and intron loss (Goddard and Burt, 1999). Homing endonucleases frequently possess maturase activity required for intron splicing. If their reading frame degenerates or is completely lost, proteins (like Mrsps) coded by nuclei and imported to mitochondria have to evolve rapidly to compensate the maturase activity loss. These splicing factors are often associated with several introns, what generates new and specific Dobzhansky–Muller pair with the potential to establish a new lineage. Introns considered as parasitic and selfish elements can thus serve as a triggers of speciation.

### Conflict of interest statement

The authors declare that the research was conducted in the absence of any commercial or financial relationships that could be construed as a potential conflict of interest.
